# Subcortical control of the default mode network: Role of the basal forebrain and implications for neuropsychiatric disorders

**DOI:** 10.1016/j.brainresbull.2022.05.005

**Published:** 2022-05-11

**Authors:** David D. Aguilar, James M. McNally

**Affiliations:** a VA Boston Healthcare System, West Roxbury, MA, USA; b Department of Psychiatry, Harvard Medical School, Boston, MA, USA

**Keywords:** Parvalbumin, Medial frontoparietal network, Cholinergic neurons, Brain networks, Functional magnetic resonance imaging, Schizophrenia

## Abstract

The precise interplay between large-scale functional neural systems throughout the brain is essential for performance of cognitive processes. In this review we focus on the default mode network (DMN), one such functional network that is active during periods of quiet wakefulness and believed to be involved in introspection and planning. Abnormalities in DMN functional connectivity and activation appear across many neuropsychiatric disorders, including schizophrenia. Recent evidence suggests subcortical regions including the basal forebrain are functionally and structurally important for regulation of DMN activity. Within the basal forebrain, subregions like the ventral pallidum may influence DMN activity and the nucleus basalis of Meynert can inhibit switching between brain networks. Interactions between DMN and other functional networks including the medial frontoparietal network (default), lateral frontoparietal network (control), midcingulo-insular network (salience), and dorsal frontoparietal network (attention) are also discussed in the context of neuropsychiatric disorders. Several subtypes of basal forebrain neurons have been identified including basal forebrain parvalbumin-containing or somatostatin-containing neurons which can regulate cortical gamma band oscillations and DMN-like behaviors, and basal forebrain cholinergic neurons which might gate access to sensory information during reinforcement learning. In this review, we explore this evidence, discuss the clinical implications on neuropsychiatric disorders, and compare neuroanatomy in the human vs rodent DMN. Finally, we address technological advancements which could help provide a more complete understanding of modulation of DMN function and describe newly identified BF therapeutic targets that could potentially help restore DMN-associated functional deficits in patients with a variety of neuropsychiatric disorders.

## 1. Introduction

The neural mechanisms which support consciousness and our engagement with the external world remain poorly understood. Such processes necessitate the precise cooperation of specialized functional networks, which must dynamically coordinate their activity and interactions as a function of task performance and cognitive demand. Accurately defining the brain regions that comprise these networks, their properties, and the mechanisms which regulate their function can provide novel targets for therapeutic interventions for a number of neurological disorders associated cognitive deficits.

One such functional network that has garnered significant interest over the last few decades is the default mode network (**DMN**), a large-scale brain circuit that is activated during quiet wakefulness and deactivated during goal-directed tasks which evoke cognitive load. This network is conserved across a number of mammalian species, including humans, nonhuman primates, cats, and rodents and generally includes the medial prefrontal cortex (**mPFC**), the posterior cingulate cortex (**PCC**) and its surrounding regions in lateral parietal and temporal cortices (Shulman et al., 1997; Buckner et al., 2008; Raichle, 2015; Nair et al., 2018) (See Table 1). This network is somewhat unique in its brain-wide connectivity and capacity for integrated information processing, especially within contexts requiring a memory-based “autopilot” behavioral role (Vatansever et al., 2017). Activation of the DMN is also increased during advanced forms of thought and has been suggested to allow people to extract themselves from a first-person perspective to evoke introspection, imagination, mental state attribution, planning, and social inferences (Anticevic et al., 2012; Buckner and DiNicola, 2019; Jenkins, 2019). The network’s equidistance from sensory and motor areas in both anatomical and functional connectivity domains allows for high levels of abstraction, sensory integration, and information processing (Margulies et al., 2016).

There are multiple functionally distinct brain networks, beyond the DMN, which compete for activation. Several functional networks have been observed to exhibit activity that is anticorrelated with the DMN, particularly during externally oriented cognition (Buckner and DiNicola, 2019). This suggests that the DMN is suppressed while these networks become active. These anticorrelated networks include the dorsal frontoparietal network (**D-FPN,** attention network), midcingulo-insular network (**M-CIN,** salience network) or the lateral frontoparietal network (**L-FPN,** control network, see Table 1 for more detail) (Anticevic et al., 2012; Uddin et al., 2019). DMN suppression has been suggested to support certain types of goal-directed cognitive process. The M-CIN may also help transition between the DMN and L-FPN to guide attention towards behaviorally or biologically relevant stimuli (Menon, 2015), while the L-FPN appears to be a top-down “functional hub” that manages interactions between the various brain networks (Marek and Dosenbach, 2018). However, DMN activity has also been observed to be important for cognition and behavior, especially within specific contexts. Certain cognitively demanding tasks that require self-referential thinking (i.e., autobiographical planning) can lead to coactivation of the DMN and L-FPN (Spreng et al., 2010) [for more examples see Spreng, 2012]. Additionally, subregions of the DMN including the precuneus demonstrate activation during externally-focused task performance (information processing speed task, high level prediction-error), perhaps acting as a transition between the DMN and task-positive networks (da Silva et al., 2020; Brandman et al., 2021; Lyu et al., 2021). A healthy balance between the activity of these brain networks appears essential as abnormally strong DMN functional connectivity or deficient DMN activation/suppression can be associated with certain neuropsychiatric disorders (Anticevic et al., 2012; Hamilton et al., 2015; Zhou et al., 2016).

Our present understanding of DMN activity is based principally on work utilizing either positron emission tomography (**PET**) or functional magnetic resonance imaging (**fMRI**) techniques (Jenkins, 2019). This almost exclusive reliance on correlational findings from neuroimaging studies can lead to varying results that are difficult to interpret. Thus, there is a need for future work focusing on understanding the properties of DMN from different levels of analysis. Below, we describe the role of abnormal DMN function in neuropsychiatric disorders with a particular focus on schizophrenia and describe recent clinical and preclinical findings which may provide novel insights into the biological mechanisms responsible of modulation of DMN activity. Finally, we discuss technological advancements that may lead to further insight into the biological mechanisms behind the DMN.

## 2. The role of the DMN in schizophrenia and other neuropsychiatric disorders

Abnormalities in DMN activity have been reported across numerous neurological and neuropsychiatric disorders (For a comprehensive review of this topic see; Whitfield-Gabrieli and Ford, 2012; Mohan et al., 2016; Hu et al., 2017; Allen et al., 2019). Here, we provide a brief discussion focusing on DMN activity in schizophrenia, a disorder where patients experience hallucinations and delusions along with generalized cognitive and social deficits. Disturbances in the DMN or in the ability to rapidly switch between functional networks can have significant consequences on cognition, attention, and memory (Whitfield-Gabrieli et al., 2009; Anticevic et al., 2015; Woodward and Heckers, 2016). Abnormal regulation of DMN and M-CIN transitions may result in salience being applied to meaningless external and internal stimuli, perhaps giving rise to hallucinations and delusions (Palaniyappan and Liddle, 2012). Indeed, hallucinations in schizophrenia are associated with sudden disengagement of the DMN and disinhibition of sensory regions, and hallucinations seem to disappear when L-FPN regions are activated (Waters et al., 2012; Jardri et al., 2013; Lefebvre et al., 2016; Leroy et al., 2017; Allen et al., 2019). Additionally, the volume of the M-CIN is associated with the severity of delusions and hallucinations in patients with schizophrenia (Crespo-Facorro et al., 2000; Palaniyappan et al., 2011).

DMN suppression facilitates certain types of goal-directed cognitive processes (Anticevic et al., 2015; Buckner and DiNicola, 2019). Patients with schizophrenia show sustained activation in DMN subregions and reduced activation in anticorrelated networks including the L-FPN during tasks involving executive function (Minzenberg et al., 2009; Nygard et al., 2012; Razavi et al., 2013; Kindler et al., 2015). Thus, an impaired ability to switch between these networks may lead to downstream cognitive impairment (Hugdahl, Loberg, et al., 2009; Hugdahl, Westerhausen, et al., 2009; Allen et al., 2019). Zhou and colleagues used resting-state fMRI approaches during a working memory task and found that within first episode schizophrenia patients, those with cognitive impairments show reduced suppression of DMN regions including the mPFC and PCC, while those without cognitive impairments do not (Zhou et al., 2016).

Despite inconsistent findings regarding network oscillation frequencies across patients with schizophrenia, NMDA receptor dysfunction may alter theta and delta rhythms among DMN and hippocampal regions which could affect working memory, network rhythms, and other schizophrenia-related phenotypes (Hunt et al., 2017). Both clinical and preclinical findings suggest that DMN cortical function, as measured by fMRI bold signal, appears to be linked to gamma band activity as measured by local field potential (Leopold et al., 2003; Fox et al., 2018). Clinical studies reveal that gamma band oscillations in DMN-associated brain regions are enhanced during quiet wakefulness, transiently suppressed during cognitive tasks in a manner that is time-locked to the initiation of task performance, and are proportional in amplitude to task difficulty (Miller et al., 2009; Dastjerdi et al., 2011; Ossandon, Jerbi et al., 2011; Ramot et al., 2012; Nair et al., 2018). Suppression of gamma oscillations in DMN regions during a sensorimotor task also occurs in nonhuman primates (Hayden, Smith et al., 2009; Pearson et al., 2011) and cats (Popa et al., 2009; Nair et al., 2018). Recent preclinical evidence supports these observations using transgenic serine racemase knockout mice, a model of NMDA receptor dysfunction that exhibits phenotypes similar to schizophrenia (Basu et al., 2009; DeVito et al., 2011; Balu et al., 2012; Puhl et al., 2019; Balla et al., 2020). Serine racemase knockout mice have recently been shown to exhibit impaired social memory (Aguilar et al., 2021). Pairing EEG recordings with social task performance revealed that the DMN-associated prefrontal/anterior cingulate cortex of these knockout mice showed elevated spontaneous broadband gamma activity just prior to social investigations. This effect was associated with suppressed task-evoked gamma-band response, likely due to impaired suppression of DMN-like spontaneous gamma band activity in this transgenic model.

Recent clinical findings have elucidated a potential mechanism behind impaired deactivation of DMN activity in psychiatric disorders. Using fMRI and magnetic resonance spectroscopy (**MRS**) approaches in healthy human adults, Gu and colleagues discovered that functional network interactions between a DMN node (PCC) and the M-CIN mediates the association between the PCC’s excitatory-inhibitory balance and task-induced deactivation of the DMN (Gu et al., 2019). This suggests that deficient DMN deactivation during task performance could have two underlying mechanisms: 1) an excitatory-inhibitory imbalance in a DMN node like the PCC (which could be rescued with pharmacological targeting), and/or 2) desensitization to long-range salience network (M-CIN) inputs on a DMN node (which could be rescued with neuromodulation and upstream circuit targeting) (Gu et al., 2019).

Beyond schizophrenia, abnormal functional connectivity within the DMN has also been associated with Alzheimer’s disease (Greicius et al., 2004). In Parkinson’s disease, enhanced DMN connectivity has been associated with hallucinations. Abnormal DMN connectivity in mood disorders can be associated with rumination, the duration and number of depressive episodes, and cognition (Mohan et al., 2016). Specifically, depressive rumination in major depressive disorder (MDD) may be caused by abnormally strong functional connectivity between the DMN and a subregion of the prefrontal cortex (Hamilton et al., 2015), while hyperactive DMN activity in MDD may be linked to cognitive impairment (Rose et al., 2006). Abnormal network connectivity exists across many networks in MDD including the DMN, salience network (M-CIN), and control network (L-FPN) (Brakowski et al., 2017). Anxiety and affective disorders are also suggested to be influenced by DMN abnormalities. Increased DMN activation may enhance anxiety and worry (Servaas et al., 2014; Gentili et al., 2015), but its relationship during task performance may depend on the task utilizing executive functions (Eysenck et al., 2007; Fales et al., 2008; Maresh et al., 2014; Allen et al., 2019).

Finally, a resting-state fMRI study of children ages 7–12 with autism spectrum disorder (ASD) revealed hyperconnectivity within the M-CIN, DMN, frontotemporal, motor, and occipital (visual) networks compared to typically developing children’s brains (Uddin et al., 2013, 2019). Specifically, posterior DMN regions including the precuneus, posterior cingulate, and left angular gyrus all showed greater functional connectivity in children with ASD compared to typically developing children, which might be associated with social and interpersonal cognition deficits (Uddin et al., 2013). Other studies have reported DMN hypoconnectivity in adults and adolescents with ASD which was correlated with the severity of their social and communication deficits, but these cohorts were slightly older (~ 11–20 years old) (Assaf et al., 2010).

## 3. Subcortical control of the DMN: role of the basal forebrain

### 3.1. Studies of subcortical DMN control in healthy human participants

Recent clinical studies have provided great leaps in our understanding of the DMN and its regulation. Given the widespread nature of DMN-associated processes, mechanisms involved in DMN modulation are likely to involve brain regions which influence neural activity in a widespread/global manner. Interestingly, several recent studies have identified subcortical regions which are functionally and structurally important for DMN function including the basal forebrain (**BF**) and thalamic subregions (Alves et al., 2019; Uddin et al., 2019; Harrison et al., 2021; Li et al., 2021). Alves and colleagues used novel optimized methods to analyze imaging data (MRI, resting state fMRI) from healthy humans to reimagine a basic anatomical map of the DMN based on functional alignment (Alves et al., 2019). This method identified highly interconnected regions as novel DMN nodes. Next, they used tractography of diffusion-weighted imaging and a graph theory analysis to characterize the structural connectivity of this DMN model and confirmed these novel additions to the DMN anatomical map. They concluded that subcortical regions including the BF, anterior thalamus, and mediodorsal thalamus should each be considered a central part of the default mode network based on their functional and structural connectivity (Alves et al., 2019). Their representation of the BF consisted of the nucleus basalis of Meynert, the diagonal band of Broca, and medial septal nuclei. There are some limitations to the study; the DMN maps compared nuclei locations with templates which did not account for individual variability, and tractography can be somewhat inaccurate. Nevertheless, the use of complementary structural and functional approaches that align with known neurochemistry create a convincing argument.

In a separate study, Li and colleagues utilized state of the art analysis of 7 T fMRI of resting state activity in a large cohort of healthy humans to more comprehensively map DMN subcortical connectivity, revealing extensive interconnections between the cortical DMN and subcortical regions beyond the BF and thalamus, including the brainstem, hypothalamus, basal ganglia, and ventral tegmental area (Li et al., 2021). Their analysis utilized a novel tensor decomposition method that improved contrast and spatial alignment, and they’ve released their subcortical DMN connectivity map in common formats for future research. Limitations of this method include its inability to account for severe spatial misalignment among subjects, and the challenge of applying this high resolution (7 T) map onto fMRI machines with lower resolution (3 T).

Another recent neuroimaging study supports these findings, and further suggests that BF and thalamic nodes have distinct influences on DMN function (Harrison et al., 2021). Here they observed that the BF was involved in driving suppression of DMN activity during transitions from resting-state to externally focused task-oriented behavior. The mediodorsal thalamus on the other hand, was observed to influence DMN activation during internally focused cognition. Dynamic causal modeling, a method of analysis that identifies the causal influences of brain regions on each other by studying interregional connections in baseline and dynamic (task) conditions, confirmed these findings (Harrison et al., 2021). Broadening the DMN to include additional subcortical regions may explain how this functional network can be involved in complex neural processes such as memory and emotional regulation (Alves et al., 2019).

Despite such findings, the influence of these subcortical brain regions on DMN activity remains controversial. Munn and colleagues have recently shown that subcortical neuromodulatory regions like the brainstem’s locus coeruleus (norepinephrine-rich region) and the BF’s nucleus basalis of Meynert (acetylcholine-rich region) could significantly influence cortical energy states and large-scale network transitions (Munn et al., 2021). Phasic bursts of locus coeruleus activity acted like a chemical catalyst, “flattening” the energy landscape required to transition between large scale brain networks, while phasic bursts of nucleus basalis of Meynert activity “elevated” the energy landscape making transitions between brain networks less likely (Munn et al., 2021). Resting-state fMRI findings revealed that the locus coeruleus became more active than the nucleus basalis of Meynert a few seconds before participants realized they were no longer paying attention, suggesting that the locus coeruleus can manipulate energy landscapes which may drive changes in conscious awareness (Munn et al., 2021). Therefore, these ascending arousal systems are likely involved in dynamic changes in brain networks and conscious awareness.

A recent series of fMRI neuroimaging experiments in human volunteers demonstrated how the anterior insular cortex appears to manage transitions between the default mode network and dorsal attention network (D-FPN) in response to sensory stimuli during conscious brain states (Huang et al., 2021). Unconscious brain states had minimal anterior insular cortex activation and no switching between the DMN and dorsal attention network in response to stimuli. Furthermore, if the anterior insular cortex was less active during the pre-stimulus baseline period, the subject was less likely to consciously notice a brief stimulus. Thus, the anterior insular cortex appears to “gate” conscious access to external sensory information (Huang et al., 2021). Notably, neither the BF nor thalamic regions were shown to have associations with these specific kinds of transitions between the DMN and dorsal attention network in this work. Thus, completely characterizing the role of such subcortical regions on functional network activity remains an area of active investigation.

### 3.2. Human vs preclinical DMN studies

Although the majority of DMN research has been performed in a clinical setting, preclinical approaches are becoming more common. Such work is critical to providing the ability to elucidate the biological mechanisms underlying the intrinsic connectivity and regulation of DMN (Gozzi and Schwarz, 2016). Beyond humans, DMN-like networks have been characterized in across a range of mammalian species including macaque (Vincent et al., 2007), rat (Lu et al., 2012) and mouse (Stafford et al., 2014; Whitesell et al., 2021) supporting the idea that more invasive, circuit level studies focused on DMN could be performed in lower level organisms.

**Fig. 1.**
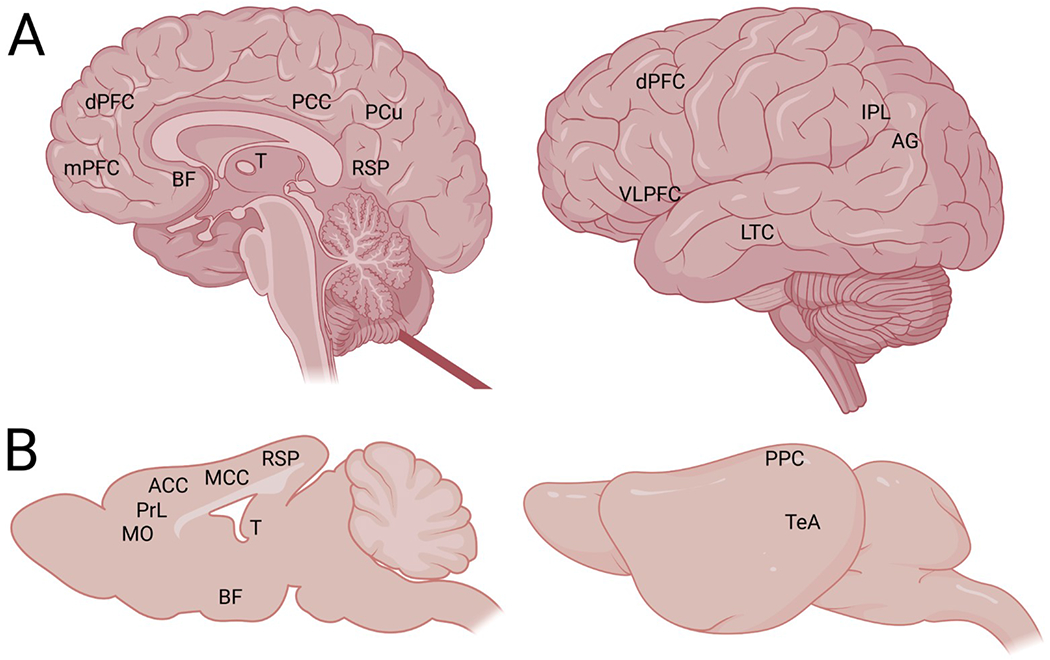
Diagram of DMN-relevant regions in human and mouse brain. Human (A) and mouse (B) brain represented as midline sagittal slice (left) and lateral view (right). Homologous areas associated with the DMN that are visible in the sagittal view (left) include: 1) the human prefrontal cortex consisting of medial prefrontal cortex (mPFC) and dorsal prefrontal cortex (dPFC) and mouse medial orbital cortex (MO), prelimbic cortex (PrL), and anterior cingulate cortex (ACC), 2) human posterior cingulate (PCC) including precuneus (PCu) and retrosplenial cortex (RSP) and mouse medial cingulate cortex (MCC) and granular/dysgranular retrosplenial cortex (RSP), 3) thalamus (T), and 4) basal forebrain (BF). Lateral view: 5) human ventrolateral prefrontal cortex (VLPFC) within the inferior frontal gyrus, 6) human lateral temporal cortex (LTC) and mouse auditory/temporal association cortex (TeA), 7) human inferior parietal lobe (IPL) which includes the angular gyms (AG) and mouse posterior parietal cortex (PPC) with secondary visual cortex. Figure created in BioRender.com.

An fMRI comparison study revealed generalized similarities across the putative rat DMN, “old world” monkey DMN, and human DMN (Lu et al., 2012). During the resting state, rats show DMN-like connectivity in the following regions (human DMN counterparts in parentheses, see Fig. 1): orbital cortex (orbital frontal cortex), prelimbic and cingulate cortex (medial prefrontal/anterior cingulate cortex), auditory/temporal association cortex (lateral temporal cortex), posterior parietal cortex including secondary visual cortex (inferior parietal lobe), granular and dysgranular retrosplenial cortex (posterior cingulate/retrosplenial cortex), and dorsal CA1 of hippocampus (hippocampus/parahippocampal cortex). Rats additionally showed a unique activation along the medial ridge of the cingulate cortex which did not appear in human brains at rest (Lu et al., 2012). Mandino and colleagues also compared resting state brain networks across human, macaque, and mouse brains by using a “triple-network” organization model that incorporates interactions between the salience (M-CIN), default mode, and central executive networks (L-FPN) to provide insight into trans-species comparisons as well as psychiatric and neurological disorders (Mandino et al., 2021). They confirmed generally homologous brain networks across these species, but the serotonin system (especially dorsal raphe) was associated with the salience network in mice instead of the DMN, as in humans. Limitations included isoflurane administration to sedate the animals into a resting state for the fMRI which could have biased the results, and the imaging resolution may not be sufficient to detect rat brain subregions with sufficient clarity (Lu et al., 2012; Mandino et al., 2021).

A recent study avoided the limitation of anesthesia in a mouse resting state fMRI study. Gutierrez-Barragan and colleagues trained C57Bl6/J mice to tolerate head-fixed resting-state fMRI scans with minimal stress and compared the functional connectivity in the awake state to other mice under halothane or isoflurane-medetomidine induced anesthesia (Gutierrez-Barragan et al., 2022). Although they observed many similarities between brain networks in awake mice and primates, some differences like a segregation of posterior vs midline DMN are not observed in higher primates (Vincent et al., 2007). This is potentially due to enhanced cortical differentiation in the primate brain compared to the rodent postero-lateral cortex (Gutierrez-Barragan et al., 2022).

Despite such findings, comparing brain networks across species is neither straightforward nor without controversy. Anatomical neurological landmarks and “connectivity fingerprints” are useful at identifying homology between species, especially in brain regions linked to common functions like movement or perception (Krubitzer, 2007; Balsters et al., 2020). In contrast, brain networks involved in higher level cognitive capabilities like the DMN and frontoparietal networks are much harder to identify across species based on their widespread physical locations and divergent cortical evolution (Xu et al., 2020). One solution is comparing functional connectivity across species engaged in a similar task. Similarities in functional organization were extracted from humans and macaques while watching movies (Mantini et al., 2012) or while at rest (Milhamet al., 2018), while using anatomical cross-species landmarks and individual comparisons to strengthen the validity of the approach (Xu et al., 2020). This work revealed greater levels of functional homology between humans and macaques in unimodal brain regions, while lower similarity was observed in systems linked to attention and more complex aspects of higher order cognition, with the most profound changes observed in the posterior DMN. These findings suggest more significant changes in DMN and other complex functional network architecture across distant species like humans and rodents.

In summary, comparing brain network activity across species is a particularly challenging task, but good experimental design can lead to impactful, informative preclinical studies. For a recent in-depth review of this topic with direct comparisons of the resting state networks and their underlying anatomy across humans, rats, and mice, please see Xu et al. (2022). For the purposes of this review, activity in the rodent anterior cingulate cortex and prelimbic cortex will be considered potentially relevant to rodent DMN activity depending on behavioral context. However, please note that this approach may be unreliable without simultaneous information from other DMN nodes. The rodent mPFC is involved in many cognitive tasks, similar to the human dorsolateral PFC, suggesting activity in PFC subregions like the anterior cingulate may represent different brain networks (DMN / M-CIN) in different contexts (Xu et al., 2022). An in-depth review of the neuroimaging literature regarding prefrontal homology between humans and rodents is outside the scope of this review, but could help clarify which prefrontal regions should and should not be considered DMN-relevant (Laubach et al., 2018; Schaeffer et al., 2020; van Heukelum et al., 2020).

### 3.3. Preclinical studies of subcortical DMN control

**Fig. 2.**
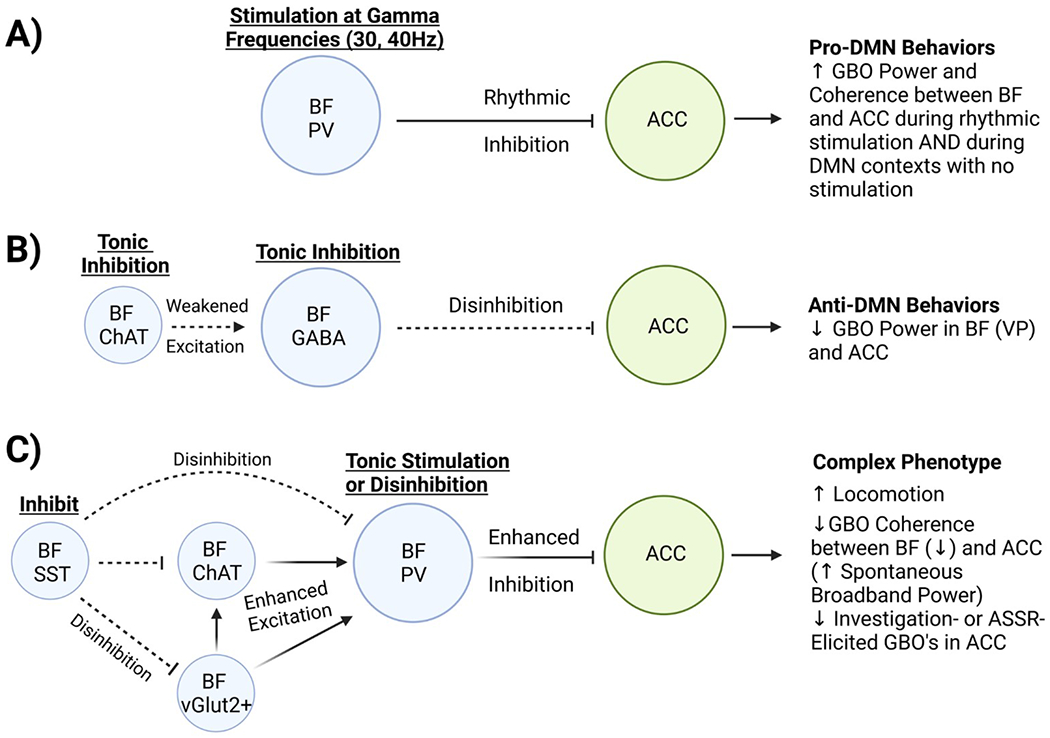
Stimulation or Inhibition of BF Cell types: Impact on DMN-like Activity and Behaviors. A) Stimulation of BF PV Neurons at gamma frequencies entrains GBO’s in the ACC and induces and maintains DMN-like behaviors including self-directed grooming, enhanced internal focus, and reduced attention to external stimuli (Lozano-Montes et al., 2020; Klaassen et al., 2021). In the absence of stimulation, DMN-like behavioral contexts induce coordinated GBO’s in the BF and ACC (Nair et al., 2018). B) Simultaneous tonic inhibition of BF cholinergic and GABAergic neurons suppresses DMN-like behaviors and reduces spontaneous GBO power in both the BF and ACC (Klaassen et al., 2021). C) Tonic stimulation of BF PV neurons (McNally et al., 2021) or disinhibition of BF neurons (unspecified cell type) through BF SST inhibition (Espinosa et al., 2019a, 2019b) has consequences that may be attributable to DMN activity. Either treatment likely results in enhanced BF PV activity due to local circuit interactions between BF cell types (Yang et al., 2017). Pro-DMN-like phenotypes include enhanced spontaneous GBO power in the ACC, reduced external task-elicited GBO’s in the ACC, and reduced novel object recognition performance suggesting impaired external attention (Espinosa et al., 2019a, 2019b; McNally et al., 2021). Anti-DMN-like phenotypes include enhanced locomotion and reduced GBO coherence between the BF and ACC. In summary, the BF appears critical for induction, maintenance, and/or suppression of DMN activity likely through specific circuitry where cortically projecting PV containing neurons play a privileged role. Figure created in BioRender.com. Abbreviations: anterior cingulate cortex (ACC), auditory steady state response (ASSR), basal forebrain (BF), cholinergic (ChAT), diagonal band (DB), default mode network (DMN), gamma-aminobutyric acid (GABA), gamma band oscillations (GBO), magnocellular preoptic area (MCPO), parvalbumin (PV), prelimbic (PrL), somatostatin (SST), ventral pallidum (VP).

Over the last few years an increasing number of preclinical studies, discussed below and featured in Fig. 2, have focused on the BF as playing a central role in modulation of DMN activity across a number of experimental modalities. The BF has been shown to be a major neuromodulatory hub, supporting broad functional connections with neocortical and subcortical brain regions critical to cognition (Lin et al., 2015; Yang et al., 2017; Zaborszky et al., 2018; Gombkoto et al., 2021). Further, degeneration of BF across age and with certain neuropathological conditions, such as Alzheimer’s disease, has been associated with both functional and cognitive impairment (Schmitz et al., 2016; Markello et al., 2018). Taken together these findings support the idea that BF plays a significant role in DMN modulation.

In macaques, unilateral inactivation of the nucleus basalis of Meynert, a BF structure with major GABAergic and cholinergic cortical projections, caused an intra-hemispheric reduction in global spontaneous fMRI signal fluctuations but did not seem to significantly affect resting-state fMRI networks including the DMN (Turchi et al., 2018). As we now understand, BF activity can decrease the likelihood of switching between networks (Harrison et al., 2021; Munn et al., 2021). Thus, inactivation of the nucleus basalis of Meynert in primates facilitated switching between the various networks during their conscious resting state activity (Turchi et al., 2018). Future experiments investigating the impact of stimulating this BF subregion on induced network transitions could be relevant for modeling aspects of schizophrenia. Nevertheless, this was an important step in determining which BF subregions are involved in DMN regulation.

In rodents, resting state fMRI data in awake mice replicated brain networks measured in anesthetized conditions but uniquely had stronger arousal-related BF connectivity with the DMN, other brain networks, and cortico-hippocampal regions (Gutierrez-Barragan et al., 2022). Awake mice also had more “cross-talk” between networks, and more regional anti-correlation (posterior vs midline DMN) compared to anesthetized states (Gutierrez-Barragan et al., 2022). This provides distinct evidence that BF and thalamic regions shape resting state fMRI network dynamics in the awake mouse, mimicking human imaging data (Alves et al., 2019). Limitations of this study include potential differences in arousal states between marginally stressed head-fixed mice and human resting state fMRI, the possibility of low doses of anesthesia causing intermittent consciousness, and the use of a single sex (male) (Gutierrez-Barragan et al., 2022). Nevertheless, this work represents one of many novel protocols for awake rodent resting state fMRI data collection which will aid the translatability of future rodent fMRI research (Jonckers et al., 2014; Ma, Ma, et al., 2018; Ma, Perez, et al., 2018; Stenroos et al., 2018; Liu et al., 2020; Gutierrez-Barragan et al., 2022).

A series of recent preclinical studies from the Rainer lab have demonstrated how BF activity influences brain states including the DMN. Using single unit and local field potential recordings in rats, they demonstrated that spontaneous gamma band oscillations in the BF’s ventral pallidum and nucleus basalis were elevated during behavior associated with increased DMN activity (e.g. quiet wakefulness and grooming) and strongly suppressed during externally directed behaviors associated with reduced DMN activity (e.g. novel object or arena exploration) (Nair et al., 2018). Further, granger causality analysis showed that spontaneous gamma band activity in the BF influenced gamma band activity in the anterior cingulate cortex, an important node of the DMN in humans and rodents (Stafford et al., 2014), and this influence was especially strong when animals were in the home cage setting (Nair et al., 2018). Together, these findings suggest that the BF plays a critical role in the regulation of DMN-like activity in mice. However, they do not speak to the precise mechanisms behind the BFs role in this process.

While the BF is generally associated with its cholinergic projections, GABAergic neurons are particularly numerous in the BF; a subpopulation of which project to the cortex (Brown and McKenna, 2015). Such long-range GABAergic projections have been suggested to play an important role in long range synchronization (Buhl and Singer, 1989; Jinno et al., 2007; Melzer et al., 2012), including those between the basal forebrain and cortex (Manns et al., 2000). A follow-up study by Lozano-Montes and company, focused on optogenetic manipulation specifically of parvalbumin (PV) expressing fast-spiking GABAergic neurons in the magnocellular preoptic area of BF in male PV-Cre rats. As shown in Fig. 2A, they observed increased behaviors relevant to DMN activity, as well as entrainment of the anterior cingulate cortex at a 30 Hz stimulation frequency (Lozano-Montes et al., 2020). Nonspecific electrical stimulation of the BF’s magnocellular preoptic area, which likely activates a combination of BF networks of PV neurons, somatostatin (**SST**)-containing GABAergic neurons, glutamatergic neurons, and cholinergic neurons, generally replicated the effects of PV-specific stimulation but also enhanced memory performance. This might be due to BF cholinergic neuron activation which can facilitate learning & memory formation (Hasselmo, 2006; Lozano-Montes et al., 2020).

Further work from this group has examined how optogenetic stimulation or inhibition of the male rat ventral pallidum, a subregion of BF, affected DMN brain states, gamma band oscillations, and learning (Klaassen et al., 2021). Here they observed that inhibition of ventral pallidum GABAergic and cholinergic cells appeared to suppress gamma oscillations in the ventral pallidum and anterior cingulate cortex (two DMN nodes) in a home cage setting, inactivate the DMN brain state, impair responses in an automatic lever-pressing task, and slightly improve acquisition during a complex attention-associated auditory task (Fig. 2B). Excitation of ventral pallidum GABAergic cells had opposite effects, enhancing gamma oscillations in the ventral pallidum and anterior cingulate cortex, and “trapping” animals in a DMN-like state of internal focus with less attention on external stimuli, which resulted in impaired acquisition of an auditory discrimination task. This suggests the ventral pallidum regulates DMN brain states to aid in instantly switching between internally (DMN) and externally (attention) guided behaviors. Together, this series of studies provide compelling evidence that the BF does in fact represent a DMN node and suggest that BF PV neurons play a role in the regulation of DMN activity. However, they remain somewhat limited, as they simultaneously inhibited GABAergic and cholinergic cells which makes it difficult to distinguish their distinct roles (Klaassen et al., 2021). Most importantly, the studies from this group rely exclusively on local field potential activity in a single cortical brain region and the BF as a proxy for DMN activity. Future studies should aim to explore neural activity in additional DMN nodes to get a more complete understanding of the strength, timing, and coordination of this directional relationship.

Similar findings were obtained in another recent study, which showed that tonic (constant low-wattage) optogenetic excitation of BF PV neurons in mice (in the diagonal band/magnocellular preoptic area) enhances broadband gamma activity in the anterior cingulate cortex (McNally et al., 2021), reminiscent of the DMN-like gamma activity reported in the Rainier group (Fig. 2C). Also, BF PV stimulation was reported to impair performance in a novel object recognition task. However, tonic BF PV stimulation also induced hyperlocomotion (McNally et al., 2021) which is not consistent with stereotypical DMN behaviors. This contradictory finding is perhaps due to the nature of the stimulation parameters utilized in this study. Such excessive stimulation of BF PV neurons may impair the ability to functionally modulate DMN-related broadband gamma activity in the cortex. This would result in excessive cortical excitation (elevated broadband gamma activity, hyperlocomotion), leading to inefficiencies in the ability to actively switch between network states, required for processing task-related sensory input.

Supporting this idea, tonic excitation of BF PV neurons in mice was observed to impair the 40 Hz auditory steady state response (ASSR) (McNally et al., 2021). The ASSR is an auditory task where the cortex entrains to the frequency of auditory stimuli. Patients with schizophrenia consistently show deficits in the 40 Hz ASSR (Kwon et al., 1999; Light et al., 2006; Brenner et al., 2009; Hirano et al., 2015; Thune et al., 2016). Selective auditory attention is important for the power of the ASSR (Manting et al., 2020). Thus, impairment of the ability to properly suppress DMN-like gamma band activity would likely impair task performance. Further supporting this idea, ventral pallidum GABAergic stimulation in rats impairs attention to external stimuli and acquisition of an auditory discrimination task (Klaassen et al., 2021). Prior work from McNally and colleagues additionally reported that optogenetic inhibition of BF PV neurons also leads to impaired 40 Hz ASSR (Kim et al., 2015) while phasic optogenetic stimulation can either enhance or decrease the ASSR depending on the relative time of presentation of optical and auditory stimuli (Hwang et al., 2019). Together, these findings suggest that appropriately timed synchronous activity of BF PV neurons can enhance ASSR responses while either inappropriate inhibition or elevation of spontaneous activity of BF PV can impair ASSR responses, illustrating the necessity of proper BF PV function to fine tune levels of DMN-like spontaneous gamma band activity for optimal cortical processing. Future studies could explore evidence of a direct connection between BF PV stimulation and DMN regulation by recording from within multiple DMN nodes with local field potential electrodes, testing additional locations for BF PV stimulation such as the ventral pallidum, and recording more behavioral characteristics associated with the DMN (grooming).

If tonic BF PV stimulation can induce phenotypes that mimic psychosis, perhaps tonic BF PV inhibition can alleviate psychotic phenotypes. Intriguingly, McNally et al. (2021) also observed that optogenetic inhibition of BF PV neurons partially rescued the elevation in cortical gamma band oscillations evoked by subanesthetic ketamine, a common method used to model aspects of schizophrenia. These experiments suggest BF PV neurons represent a promising therapeutic target in conditions like schizophrenia where DMN regulation of cortical gamma oscillations has gone awry, with the potential to improve DMN suppression and task-based switching between network states.

PV-containing neurons are not the only BF neuron subtype that can influence DMN-like brain states or behaviors. BF SST neuron manipulation also has wide-reaching consequences impacting cortical activity in DMN-relevant regions and inducing changes between DMN-like and arousal-like behaviors and brain states. Espinosa and colleagues used optogenetic approaches to inactivate BF SST neurons in transgenic mice during quiet resting state conditions and found that prelimbic cortex single unit activity was enhanced, while prelimbic coherence between single and multi-unit activity was reduced in frequencies below 10 Hz (Espinosa et al., 2019b). BF SST inhibition also enhanced the power of prelimbic low gamma band oscillations (20–40 Hz), reduced the power of prelimbic slow oscillations (0.5–1 Hz), and enhanced locomotor activity (Espinosa et al., 2019b). Importantly, as show in Fig. 2C, the influence of BF SST activity appears to be mediated via inhibition of local BF cell types (PV, cholinergic & glutamatergic), suppressing the synaptic inputs to those cell types in a format conducive to sleep, while reduced BF SST activity can increase synaptic activity in the cortex and will likely have similar results in other targets of the BF (Do et al., 2016; Espinosa et al., 2019b).

In a related study, Espinosa and colleagues recorded local field potential and single unit activity in two BF regions, the medial septum and ventral pallidum, in transgenic mice. They discovered that optogenetic inhibition of ventral pallidum SST neurons reduced ventral pallidum gamma oscillations in anesthetized mice and enhanced locomotion in conscious mice, while optogenetic inhibition of medial septum SST neurons had no effects on local BF gamma oscillations but reduced spatial memory in the Y maze task (Espinosa et al., 2019a). This and their previous work (Espinosa et al., 2019b) suggests that ventral pallidum SST activity is positively correlated with local BF gamma band oscillation activity and negatively correlated with prefrontal gamma band oscillation activity, making ventral pallidal SST neurons a key component of BF control of the DMN. Future studies should replicate this work in conscious mice to avoid the confounds of anesthesia on brain states and SST neuronal activity, and test SST activation in addition to inhibition.

By using fiber photometry to study the activity of BF cholinergic and GABAergic neurons in transgenic VGAT-Cre mice, Hanson et al. demonstrated how these signals change on a rapid time scale during an olfactory-dependent go/no-go task (Hanson et al., 2021). Specifically, BF cholinergic activity is enhanced during reward seeking behaviors and is subsequently suppressed by reward delivery, potentially signaling reinforcement. BF GABAergic activity was enhanced nonspecifically during reward seeking and non-reward seeking behaviors and suppressed by reward delivery. This study helps us understand how local cholinergic and GABAergic signaling in the BF may influence top-down regulation of sensory processing to affect reward-seeking behaviors and positive reinforcement (Hanson et al., 2021). This study adds additional context to the cell type-specific mechanisms of BF control of the DMN network.

In anesthetized transgenic rats undergoing resting state fMRI scans, unilateral chemogenetic stimulation of BF cholinergic neurons reduced functional connectivity and resting state neural activity within intra-hemispheric DMN-associated regions (Peeters et al., 2020). However, functional connectivity was not significantly lateralized to the contra-hemispheric DMN in many specific subregions (Peeters et al., 2020). Therefore, BF cholinergic activation seemed to suppress the global functional connectivity of the DMN network within a hemisphere, rather than strongly suppress connectivity within a few DMN subregions. Although these rats were neither conscious nor freely behaving, the authors believe that BF cholinergic activation may be an underlying mechanism of DMN suppression during tasks and attention (Peeters et al., 2020).

Finally, we note that the BF has been shown to have reciprocal connectivity with the insular cortex (Do et al., 2016; Zaborszky et al., 1997), a cortical brain region that plays a central role in the salience (M-CIN) network. While there has been minimal preclinical work examining the functional aspects of the connection been these regions, this places the BF in privileged position to regulate rapid dynamic switching between internally focused brain states (DMN) to externally focus states (M-CIN). Taken together, the above preclinical findings strongly support the idea that BF plays an important role in regulation of DMN function. However, there is still a great deal of work that remains to be done to definitively prove this hypothesis.

Future studies should include precise messaging about subregions of the BF or anterior cingulate cortex that are being targeted to improve rigor and reproducibility. These regions are also involved in attention, learning, and plasticity in addition to their relevance to the DMN, indicating involvement of other overlapping brain networks. Cholinergic neurons in posterior BF regions (nucleus basalis Meynert) are functionally connected with salience (M-CIN) and attention networks (D-FPN) in humans, while cholinergic neurons in anterior BF regions (medial septum, diagonal band of Broca) are functionally connected with default mode and episodic memory networks (Fritz et al., 2019). These data mimic findings based on lesion studies and axonal tracing studies in animal models (Zaborszky et al., 2015). Experiments that target distinct cell populations can help distinguish the BF’s modulation of DMN vs other brain networks and behaviors [see Lozano-Montes et al., 2020]. Subregions of the anterior cingulate cortex (orbital cortex, prelimbic cortex) are part of the rodent’s DMN and salience (M-CIN) networks (Tsai et al., 2020) and these neurons can be persistently active during periods of sustained attention in rats (Wu et al., 2017) which is uncharacteristic of a DMN mindset. Imaging or electrophysiological data from multiple DMN nodes along with behavioral data would help confirm whether activation in the anterior cingulate cortex is indeed associated with the DMN.

### 3.4. Clinical implications of subcortical DMN control

Subcortical regions including the BF and thalamic subregions (anterior thalamus, mediodorsal thalamus) can regulate DMN activity, may be considered novel DMN nodes, and are relevant to the pathophysiology of schizophrenia. Schizophrenia-related abnormalities in the mediodorsal thalamus, a region important for information processing and communication with the prefrontal cortex, have been confirmed in postmortem brains, imaging, and lesion studies (Karimi et al., 2021). Schizophrenia patients have aberrant thalamocortical functional connectivity and exhibit structural alterations in the anterior and mediodorsal thalamus (Steullet, 2020). Disrupting mediodorsal thalamic activity through chemogenetics, optogenetics, or by reducing local synaptic strength induces schizophrenia-like phenotypes in mice (Karimi et al., 2021).

BF structure or function is also implicated in neuropsychiatric conditions. BF cholinergic nuclei have lower volumes in the brains of schizophrenia patients compared to healthy controls, which is associated with attentional deficits (Avram et al., 2021). Tonic stimulation of basal forebrain parvalbumin-containing neurons in mice induces electrophysiological and behavioral phenotypes that mimic schizophrenia (McNally et al., 2021). The ventral tegmental area is another subcortical region with extensive DMN connections and relevance to psychosis (Li et al., 2021). Changes in midbrain dopamine neuron activity underlie psychotic symptoms in schizophrenia patients, but there is no pathophysiology within the dopamine neurons themselves. Instead, aberrant regulation of midbrain dopaminergic regions occurs through upstream circuits which includes BF regions (nucleus accumbens, ventral pallidum, medial septum) and thalamic nuclei (Sonnenschein et al., 2020).

In summary, BF and thalamic subregions recently implicated in DMN control are also associated with the pathophysiology of schizophrenia, making them promising therapeutic targets. Abnormalities in structure, function, or activity within these regions may disrupt brain network activity in addition to inducing neuropsychiatric phenotypes. Conversely, restoring normal function in one or more of these key DMN nodes might simultaneously improve DMN function and alleviate psychotic phenotypes. For example, reducing hippocampal-driven inhibition on the ventral pallidum can alleviate a hyperdopaminergic phenotype in a rat model of schizophrenia (Aguilar et al., 2014), and activating or deactivating the ventral pallidum can engage or release DMN-like behavioral states in rats (Klaassen et al., 2021). This approach might have cascading therapeutic effects due to the brain-wide connectivity of the DMN, its importance in sensory and information processing, and its numerous interactions with other brain networks relevant to attention, cognition, and salience.

### 3.5. Non-fMRI approaches making characterization of DMN activity more accessible

Although fMRI continues to be the most common way to study changes in DMN activity, new techniques are making DMN research more accessible, particularly at the preclinical level. While resting state MRI studies in rodents have observed patterns of intrinsic correlation between DMN associated brain regions, it has been difficult to determine if rodent DMN-like activity can be suppressed during externally oriented task performance. Changes in neural oscillations correlate with fMRI bold measures, and can be used as a putative measure of activity to detect changes in DMN-relevant regions (Leopold et al., 2003; Fox et al., 2018; Nair et al., 2018). Fakhraei et al. recently demonstrated how the rodent DMN can be studied using arrays of local field potential electrodes targeting multiple regions associated with DMN and other functional networks. These studies observed oscillatory activity within alpha and low beta frequencies (8–20 Hz) in DMN-associated regions that was correlated with activity in other DMN-associated regions. Further, they employed a novel visual stimuli based “go/wait” operant task along with distributed field potential measurements to show that this activity exhibits task related modulation in a manner similar to that described in human DMN studies (Fakhraei et al., 2021). This multi-site approach is likely to more accurately characterize functional networks such as DMN, and paves the way for future studies probing the circuit level function of DMN.

Functional ultrasound (**fUS**) is a technique that utilizes ultrasonic waves to visualize blood flow throughout the brain with great temporal and spatial precision, allowing users to detect regions of brain activation and functional connectivity similar to fMRI. Ferrier and colleagues have used this technique in lightly sedated mice to demonstrate how changes in DMN activation can be studied without fMRI. Unilateral whisker stimulation activated the contralateral barrel cortex as expected, but also suppressed activity and interhemispheric correlation within the DMN-associated retrosplenial cortex (Ferrier et al., 2020). This new technology has the capability to make rodent imaging more portable and accessible. Further, it can be performed in freely behaving animals, and be combined with other experimental modalities (e.g. optogenetics, electrophysiology). While current issues with motion artifacts (Ferrier et al., 2020) may hinder early widespread adoption, this technology provides a novel and powerful means to elucidate the mechanisms behind modulation of functional connectivity for networks including DMN.

## 4. Conclusion

In summation, the DMN and its interplay with other functional networks are likely responsible for maintaining the neural framework allowing appropriate oscillatory activity for higher cognitive function which gives rise to conscious experience (Jerath and Crawford, 2015). The literature regarding regulation of the DMN has evolved rapidly over the last few years, to the point where the DMN now includes subcortical structures like the BF. A great deal of exciting experimental work is being done at both the clinical and preclinical level, and new technologies and approaches are making it easier to explore these questions without requiring an fMRI and a well-behaved conscious animal. Many promising therapeutic targets have been identified including BF PV and BF SST neurons which can regulate cortical gamma band oscillations and DMN-like behaviors (Espinosa et al., 2019b; Lozano-Montes et al., 2020; McNally et al., 2021), BF cholinergic and GABAergic neurons which might gate access to sensory information during reinforcement learning (Hanson et al., 2021), and specific BF subregions including the ventral pallidum (Espinosa et al., 2019a) and nucleus basalis of Meynert (Munn et al., 2021). A testable hypothesis about the mechanisms underlying the DMN deactivation deficit in psychiatric disorders will also influence future studies (Gu et al., 2019). Precision medicine may allow patients to be treated based on their specific functional deficits and rescuing the role of the DMN and its interactions with other brain networks may alleviate cognitive deficits and other maladies in a variety of neuropsychiatric conditions.

## Figures and Tables

**Table 1:** Summary of relevant brain networks.

**Default mode network (DMN)** – Internal, introspective information processing (autobiographical memories, daydreaming, planning) that is suppressed during external stimulus-driven cognitive tasks and anticorrelated with networks relevant to cognitive control (lateral frontoparietal network and the midcingulo-insular network) (Anticevic et al., 2012). Major regions include the medial prefrontal cortex, posterior cingulate cortex and angular gyrus, while minor regions may include the inferior frontal gyrus, anterolateral middle temporal cortex, and posteromedial cortex (precuneus/retrosplenial) (Uddin et al., 2019; Smallwood et al., 2021). Also known as the *medial frontoparietal network* (Uddin et al., 2019).
**Lateral frontoparietal network (L-FPN)** – Control network which can initiate and flexibly adjust cognitive control by managing information processing and the activation of other networks (Marek and Dosenbach, 2018). Relevant for cognition, working memory, and task-switching (Uddin et al., 2019). Major regions include the lateral prefrontal cortex, anterior inferior parietal lobule, and intraparietal sulcus, while minor regions may include subregions of the inferior temporal lobe, cingulate gyrus, precuneus, thalamus, and caudate (Uddin et al., 2019). Also known as *central executive network, executive/frontoparietal/cognitive control network, and the extrinsic mode network* (Uddin et al., 2019).
**Midcingulo-insular network (M-CIN)** – Salience network relevant for identifying and directing attention towards important or salient information, sometimes integrating information from external sensations with internal thoughts, goals, and plans (Palaniyappan and Liddle, 2012; Uddin et al., 2019). Facilitates switching between the DMN and task-related networks to update prediction models and prepare behavioral responses (Palaniyappan and Liddle, 2012). Can manage sensorimotor functions to flexibly maintain cognitive control of goal-directed behaviors (Buckner et al., 2008; Anticevic et al., 2012; Marek and Dosenbach, 2018). The major regions include the bilateral anterior insula and anterior midcingulate cortex, while minor regions may include the inferior parietal cortex, right temporal parietal junction, lateral prefrontal cortex, and various subcortical regions (Uddin, Yeo et al., 2019). Also known as *salience network, cingulo-opercular network, ventral attention network*.
**Dorsal Frontoparietal network (D-FPN)** – Visuospatial attention network which can prime and focus attention on external stimuli and responses (Corbetta and Shulman, 2002; Anticevic et al., 2012; Buckner and DiNicola, 2019; Uddin et al., 2019). Major regions include superior parietal lobule, intraparietal sulcus, middle temporal complex, frontal eye fields, while minor regions include ventral premotor cortex, right dorsolateral prefrontal cortex, superior colliculus (Shulman et al., 1997; Anticevic et al., 2012; Uddin et al., 2019). Also known as *dorsal attention network, dorsal attention system*.
